# Novel loss-of-function *PRRT2* mutation causes paroxysmal kinesigenic dyskinesia in a Han Chinese family

**DOI:** 10.1186/1471-2377-14-146

**Published:** 2014-07-16

**Authors:** Zhisong Ji, Quanxi Su, Lingling Hu, Qi Yang, Cuixian Liu, Jun Xiong, Fu Xiong

**Affiliations:** 1 Deparment of Medical Genetics, School of Basic Medicine Sciences, Southern Medical University, Guangzhou, China; 2 Department of Neurology, Yunfu People’s Hospital, Yunfu, China

**Keywords:** Sanger sequencing, Novel mutation, c.186-187delGC, Protein function, PKD, *PRRT2*

## Abstract

**Background:**

Mutations in proline-rich transmembrane protein 2 (*PRRT2*) are a cause of paroxysmal kinesigenic dyskinesia (PKD). In this study, we investigated the *PRRT2* gene mutation in a Chinese Han family with PKD and study the pathogenesis of the mutation with *PRRT2* gene.

**Methods:**

Peripheral venous blood was taken from the family members. Sanger sequencing was used for novel mutation sequencing. For the pathogenesis with the novel mutation was analyzed by bioinformatics, real-time PCR, subcellular localization and Western blot.

**Results:**

The Sanger sequencing showed a novel mutation, c.186-187delGC, a deletion mutation, in exon 2 of the *PRRT2* gene, the frameshift mutation generated a truncated protein that was stably expressed in transfected Human embryonic kidney (HEK) 293 cells. A subcellular localization assay in COS-7 cells with GFP-tagged protein showed nuclear localization for the mutant protein while the wild-type protein was localized in membranes. Co-transfection of HEK293 cells with wild-type and mutant expression plasmids cells did not influence mRNA or protein expression from the wild-type plasmid.

**Conclusions:**

Our findings demonstrated that the c.186-187delGC mutation resulted in a truncated protein from the *PRRT2* gene to involve in PKD pathogenesis with haploinsufficiency. The results extend the mutation spectrum of the *PRRT2* gene and provide a new example for studying the pathogenesis of the mutated *PRRT2* gene.

## Background

Paroxysmal kinesigenic dyskinesia (PKD) is a rare autosomal dominant neurological disorder with childhood or early adulthood onset. PKD is characterized by recurrent, brief attacks of involuntary movement that are usually triggered by sudden voluntary movement [1-3]. Most idiopathic PKD patients have a family history of PKD, usually inherited in an autosomal dominant pattern [4]. PKD often improves with age and most patients show a favorable response to anticonvulsant medications, particularly carbamazepine or phenytoin [4,5].

Proline-rich transmembrane protein 2 (*PRRT2*) is a causative gene for PKDs [6,7]. We investigated a Han Chinese family with PKD and found the novel *PRRT2* mutation c.186-187delGC that was heterozygous in the family. We investigated the function and potential pathogenic mechanism of the mutated gene. We found that the novel mutation was a loss-of-function mutation in *PRRT2* that caused PKD by haploinsufficiency. Our results could be a reference model for studying the potential pathogenic mechanism of PKD caused by variations in the *PRRT2* gene.

## Methods

### Patients

**Figure 1:**
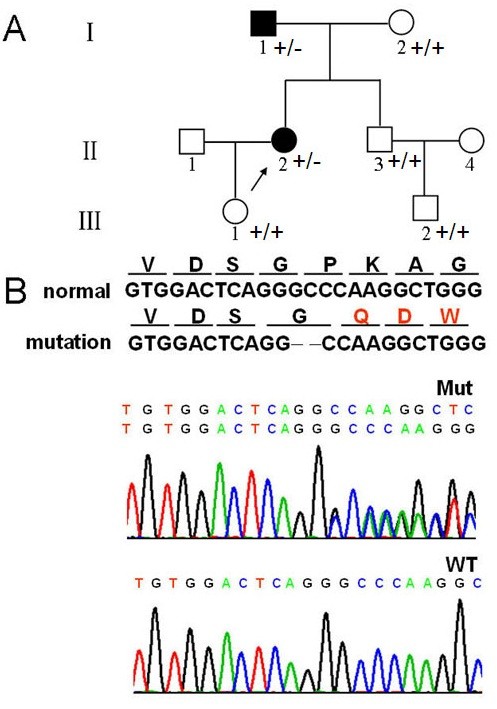
**Family pedigree and mutation screening. A**. Family pedigree. Arrow, proband; I-1, relative with the mutation who had a similar attack with light symptoms several times in youth with recovery without drugs; **B**. Mutation screening. Sequences of mutation (middle) and wild-type (lower). Red (upper), amino acid differences between mutation and wild-type.

The study was approved by the Southern Medical University ethics committee. Patients and relatives analyzed in the study gave signed informed consent before inclusion. Patients were recruited from outpatient clinics. The proband (II-2), a 45-year-old woman, was referred to the Department of Neurology at Yunfu People’s Hospital for consultation about involuntary movements. Clinical examination of the proband was performed by an experienced neurologist. Medical and neurologic histories were obtained from 8 family members (Figure 1A). The 200 normal controls matched by gender and ethnic origin were selected and recruited from healthy individuals from the neurology clinics at Yunfu People’s Hospital.

### Mutation screening

A standardized phenol/chloroform extraction method was performed to extract genomic DNA from peripheral blood. Polymerase chain reaction (PCR) primers were designed using Primer3 (http://frodo.wi.mit.edu/) to cover the four *PRRT2* exons. Appropriate annealing temperatures were selected for PCR. PCR products were analyzed with agarose gel electrophoresis and used for Sanger sequencing. This mutation that c.186-187delGC was not detected in 200 healthy volunteers by high-resolution melting analysis.

### Bioinformatics

Three-dimensional structures of wild-type and mutant *PPRT2* proteins were predicted with I-TASSER, and functional effects of the mutant protein were estimated with SOSUI.

### Cell localization studies

The subcellular localization of the *PRRT2* protein and the impact of the mutation was studied. COS-7 cells were transfected with wild-type or mutant *PRRT2* plasmids. At 48 hours after transfection, cells were rinsed three times with PBS and nuclei were stained with 4′, 6-diamidino-2-phenylindole (DAPI; Sigma, St. Louis, MO) and viewed by fluorescence microscope (Nikon, Eclipse Ti-U, Tokyo, Japan). The wild-type *PRRT2* gene was cloned into the *Hin*dIII and *Bam*HI sites of pEGFP-C1 from GENEWIZ(GENEWIZ, Inc. ,South Plainfield, New Jersey, USA) and used as the backbone for cloning the mutant form of *PRRT2*, ∆c*PRRT2*-pEGFP-C1. Mutant *PRRT2* was cloned by site-directed mutagenesis via QuikChange Site-Directed Mutagenesis Kit (STRATAGENE). Point-mutation primers were: primer-F, 5′- CCTGTGGACTCAGGCCAAGGCTGGGCT-3′ and primer-R, 5′-CCAGCCCAGCCTTGGCCTGAGTCCACAGGG-3′.

### RNA analysis

Human embryonic kidney (HEK) 293 cells were cultured in DMEM (Invitrogen) supplemented with 10% fetal bovine serum. Recombinant plasmids with wild-type or mutant *PRRT2* genes were constructed and transfected into HEK293 cells using Lipofectamine 2000 (Invitrogen) according to the manufacturer’s instructions. Total RNA was isolated from transfected HEK293 cells. PrimeScript RT-PCR kits (TaKaRa Biotechnology, Dalian, Co., Ltd) were used to synthesize complementary DNA. SYBR Green (Bio-Rad Laboratories, California, USA)-based relative quantitative RT-PCR was used to measure mRNA from wild-type and mutant *PRRT2* alleles. Differences in mRNA levels between wild-type and mutant alleles was assessed by a two-standard-curves method. Signal intensity from the *PRRT2* gene was normalized to the β-actin gene. The standard curve was the Ct value plotted against the log of the input mRNA concentration at five 10-fold serial dilutions. Three-step real-time PCR analysis generated two standard curves for linear quantitation of mRNA, with y = -3.169x + 15.08 (R2 = 0.999) for *PRRT2* mRNA and y = -3.182x + 13.7 (R2 = 0.996) for β-actin mRNA. The primers of both wild-type and mutant type: Forward (5′- > 3′): CCAGAAACCTCGGGACTACA and Reverse (5′- > 3′): CTGTTCCGGGACATGACAG. Gene expression levels were calculated with the (2^–ΔΔCT^) method in SYBR Green system. Target-gene expression was normalized to β-actin expression. All real-time RT-PCR reactions were performed in triplicate, and results obtained from three reactions were used for calculating mean values and standard deviations. Dissociation curve analysis was carried out after PCR amplification to confirm the absence of non-specific amplification products and primer dimers. The result indicated that mRNA expression level of mutant type was double of that in wild-type.

### *PRRT2* protein in HEK293 cells

HEK 293 cells were transfected with pEGFP-C1 producing a ∆c*PRRT2* fusion protein or wild-type *PRRT2* to measure protein expression from the wild-type or mutant genes. Cells were harvested 24 h after transfection. To study the influence of ∆c*PRRT2* protein on the wild-type *PRRT2* protein, wild-type and mutant plasmids were co-transfected into HEK293 cells with 2 μg of wild-type plasmid and 2 μg, 1.5 μg, 1 μg or 0.5 μg mutant plasmid. EGFP-C1 vector was used to balance the dose to 0, 0.5 μg, 1 μg or 1.5 μg. Cells were homogenized in RIPA buffer with Protease Inhibitor Cocktail (Sigma) and blots were incubated with rabbit anti-GFP (1:1000, Santa Cruz) overnight at 4°C, then goat anti-rabbit IgG-HRP (1:5000, Santa Cruz) at room temperature for 1 hr, then detected with Immobilon Western Chemiluminescent HRP substrate (Millipore).

## Results

### Clinical features

The patient had a detailed clinical evaluation by an experienced neurologist, including age and gender, age at onset, attack frequency, duration of attack, triggers, affected limbs and response to anticonvulsant. Clinical features of the proband and her family members are in Table 1. The proband (II-2) experienced onset at the age of 10 with unilateral or bilateral paroxysmal choreiform movement (with hunch), triggered by sudden activity such as standing or walking, lasting less than one minute and then alleviating. Initiation of attacks was once or twice a month, gradually increasing in frequently to 4 to 5 times a day. Valproic acid was ineffective, but carbamazepine decreased attacks. After the age of 18 years, attacks were rare and the patient no longer took medicine. The proband’s father (I-1), a 76-year-old man, had several similar attacks when he was younger, with lighter symptoms and recovery without drugs. Other details of clinical presentation were unclear.

**Table 1:** Clinical features and genotypes of the patient and family

**Subject**	**PRRT2 mutation**	**Protein alteration**	**Age/Gender**	**Age at onset**	**Attack frequency**	**Duration of attack**	**Triggers**	**Affected limbs**	**Response to anticonvulsant**
II-2	c.186-187delGC	P63Qfs*70	45/F	10	4-5/d	<1 min	SM	Right/left/bilateral	Carbamazepine
I-1	c.186-187delGC	P63Qfs*70	76/M	No	U	U	U	U	No
I-2	No	No	68/F	-	-	-	-	-	-
II-1	NA	NA	44/M	-	-	-	-	-	-
II-3	No	No	41/M	-	-	-	-	-	-
II-4	NA	NA	40/F	-	-	-	-	-	-
III-2	No	No	13/M	-	-	-	-	-	-
III-1	No	No	18/F	-	-	-	-	-	-

F: female; M: male; U: unknown; SM: sudden movement; NA: not available.

### Identification of *PRRT2* mutations

**Figure 2:**
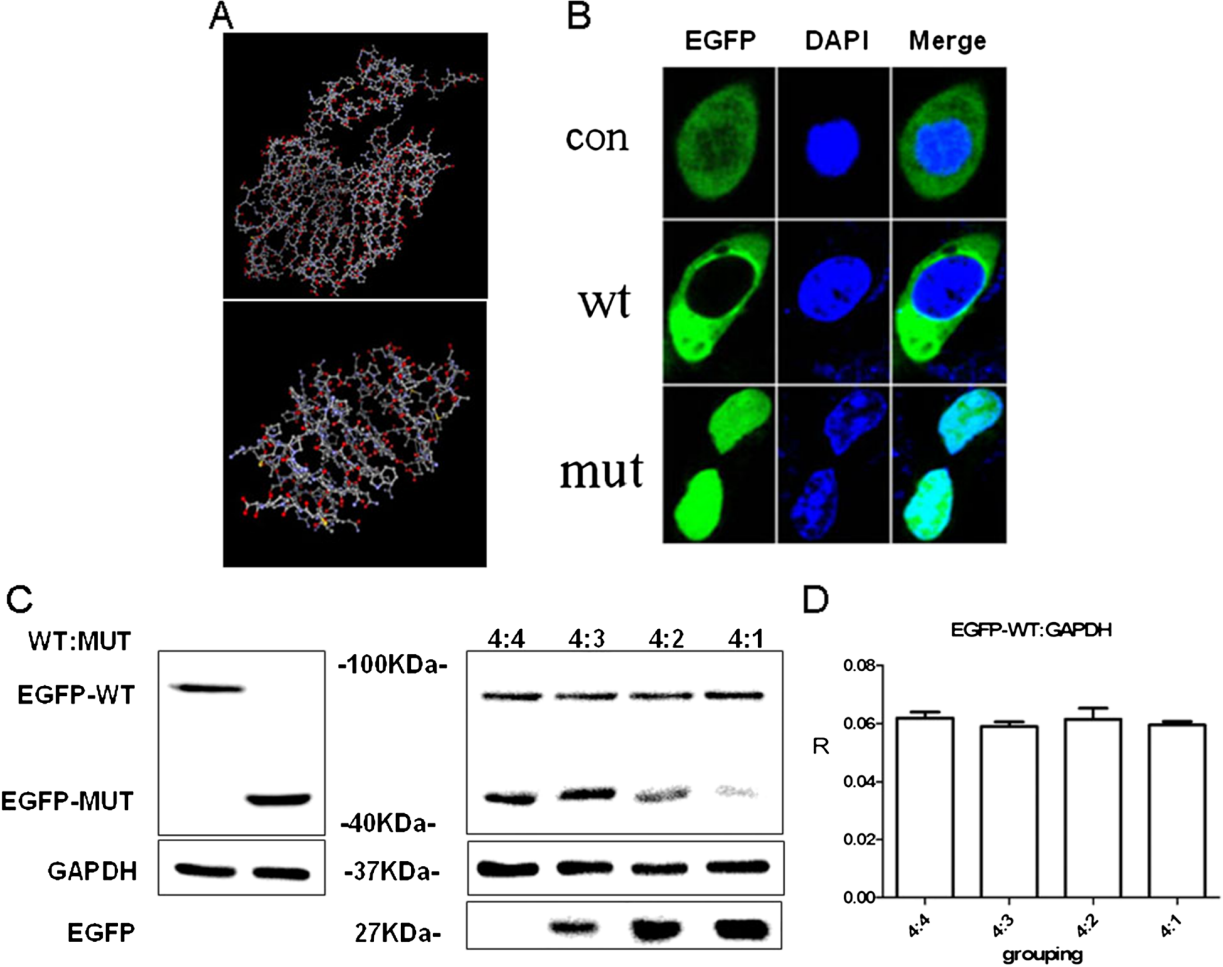
**The function study with the mutated protein. A**. Predicted protein structure: wild-type (upper) and mutant (lower) by I-TASSER. **B**. COS7 cells with cellular location of wild-type and mutant. **C**. Wild-type and mutant protein by western blot. Left, HEK293 cells transfected with GFP-tagged PRRT2 gave a ~90 kDa band; EGFP-fusion truncated mutant gave a ~45 kDa band. Right, HEK293 cells co-transfected with 2 μg wild-type plasmid and 2 μg, 1.5 μg, 1 μg or 0.5 μg mutant plasmid showed no difference in wild-type PRRT2 protein. EGFP, 27 kDa; GAPDH 37 kDa. **D**. No significant difference was seen in protein expression for co-transfected wild-type and mutant plasmids at different ratios.

DNA sequencing revealed a deletion of two nucleotides (GC) from codon 186 to codon 187 in the proband and her father’s genomic DNA samples (Figure 1B). The deletion created a stop codon (TGA) that resulted in premature termination of translation at codon 398 for a truncated protein of 132 amino acids. Prediction using SOSUI (http://www.bionity.com/en/encyclopedia/SOSUI/) software indicated that the premature termination code led to the loss of transmembrane function (Figure 2A) and changed the tertiary structure and function of the *PRRT2* protein.

### Functional analysis

Results showed that expression of the mutant was higher than expression of the wild-type. We also found differences in the localization of wild-type and mutant protein. *PRRT2* has 4 exons encoding 340 amino acids and is predicted to have two transmembrane domains (amino acids 269–289 and 318–338) [8]. The subcellular localization of wild-type protein from pEGFP-C1-*PRRT2* was assayed in COS-7 cells. The protein from ∆c*PRRT2*-pEGFP-C1 was located in the nucleus. Thus, the truncated protein had a different subcellular localization (Figure 2B). An identically sized band (~90 kDa) was observed from cells expressing the GFP-tagged wild-type *PRRT2* fusion protein. The GPF-tagged ∆c*PRRT2* fusion protein showed a band of ~45 kDa (Figure 2C). We did not detect any obvious differences in the mRNA or protein when a wild-type *PRRT2* plasmid was co-transfected with different amounts of a plasmid expressing mutant *PRRT2* (Figure 2C, 2D).

## Discussion

PKD is a autosomal dominant hereditary disease with clinical heterogeneity, such as the age of onset, which is typically in childhood or adolescence; range, which is 4 months to 57 years; and clinical features of patients, which varies among individuals, even in the same family [3]. In this case, the proband harbored a novel mutation from her father, however, her father had almost no clinical symptoms. This example further demonstrated the heterogeneity of the PKD phenotype. Other genetic factors such as pleiotropy might be further analyzed to elucidate the heterogeneity.

*PRRT2* has been widely investigated as a causative gene of PKD and many mutations in the *PRRT2* gene are associated with PKD [9]. In the early literature, *PRRT2* mutations in PKD patients are in three general molecular groups: a) single-base deletions, duplications or substitutions leading to premature termination [10]; b) missense mutations; and c) microdeletions leading to frameshifts. We found a novel mutation (c.186-187delGC) that was a deletion of two nucleotides, GC, in the coding region of exon 2. The mutation caused a frameshift leading to a premature termination at codon 398. *PRRT2* has 4 exons encoding 340 amino acids and the protein is predicted to have two transmembrane domains of amino acids 269–289 and 318–338 [8]. However, the truncated protein lacked a proline-rich domain and two transmembrane domains, which might have caused loss of protein function and resulted in location of the protein in the nucleus [11]. Functional analysis of the truncated protein showed that the mutation did not influence expression. Expression with the wild-type protein indicated that the pathogenic mechanism did not have a dominant negative effect. We think the overexpress of the mutation type will do nothing on the wild type protein by an artificial assay. Based on the experiments performed in vitro, we think that haploinsufficiency is not disputed. And nonsense mediated decay is one of possible mechanism to haploinsufficiency [12], the mechanism of this mutation needs further investigation. The cause for pathology was loss of function of the mutation protein with the normal protein unable to sustain normal functions, resulting in haploinsufficiency and more research should be done to understand the mechanism. PDK is a paroxysmal neurological disorder disease with a favorable response to the ion-channel blocker carbamazepine at a low dose. PKD is believed to be an ion channelopathy [13]. As a transmembrane protein, *PRRT2* might be involved in ion channels or regulation of ion channels related to PKD [14]. The novel mutant protein was located in the nucleus and might influence on the ion channels loss of function.

## Conclusion

In conclusion, we found a novel mutation that resulted in a truncated protein from the *PRRT2* gene that was involved in PKD pathogenesis. We performed functional analysis of the novel mutation to elucidate the pathogenic mechanism and haploinsufficiency. Our results extend the mutation spectrum of the *PRRT2* gene of PKD. With additional research, the potential mechanism associated with this disease may be further understanding.

## Abbreviations

*PRRT2*: Proline-rich transmembrane protein 2; PKD: Paroxysmal kinesigenic dyskinesia; HEK: Human embryonic kidney.

## Competing interests

The authors declare that they have no competing interests.

## Authors’ contributions

FX contributed substantially to conception and design. QXS participated in the acquisition of clinical sample and clinical examination. ZSJ carried out the molecular genetic studies, participated in the sequence alignment and drafted the manuscript. LLH, QY CXL, JX participated in the sequence alignment, culture cells and helped to draft the manuscript. All authors critically revised the article for important intellectual contents, and approved the final manuscript.

## Pre-publication history

The pre-publication history for this paper can be accessed here:

http://www.biomedcentral.com/1471-2377/14/146/prepub
